# Electrochemical Activity and Damage of Single Carbon Fiber

**DOI:** 10.3390/ma14071758

**Published:** 2021-04-02

**Authors:** Xiaodong Chen, Chi Zhang, Guang-Ling Song, Dajiang Zheng, Yang Guo, Xiaosong Huang

**Affiliations:** 1 Center for Marine Materials Corrosion and Protection, College of Materials, Xiamen University, 422th. Siming Rd, Xiamen 361005, China; 20720171150004@stu.xmu.edu.cn (X.C.); 13520735172@163.com (C.Z.); zhengdajiang@xmu.edu.cn (D.Z.); 2 State Key Laboratory of Physical Chemistry of Solid Surfaces, Department of Chemistry, College of Chemistry and Chemical Engineering, Xiamen University, 422th. Siming Rd, Xiamen 361005, China; 3 School of Mechanical and Mining Engineering, The University of Queensland, Brisbane, QLD 4072, Australia; 4 Advanced Materials, China Science Lab, GM Global R&D, 56th. Jinwan Rd, Pudong, Shanghai 200120, China; yang.guo@novelis.com; 5 Chemical and Materials Systems Lab, GM Global R&D, 30500 Mound Rd., Warren, MI 48090, USA; xiaosong.huang@gm.com

**Keywords:** carbon fiber, surface, electrochemistry, microstructure, degradation

## Abstract

The electrochemical activity of a carbon fiber was characterized at different potentials in 3.5 wt.% NaCl solution, and the fiber cylindrical surface changed by polarization at different potentials was revealed by SEM, AFM, optical microscopy, FTIR spectroscopy, Raman spectroscopy, and XRD. The results showed that the carbon fiber exhibited different electrochemical activities at some polarization potentials; within a 3V potential range the anodic and cathodic polarization current densities stepped up by more than 5 orders of magnitude, and the carbon fiber (CF) surface dramatically changed with time. Strong anodic polarization appeared to facilitate the breakdown of C-C covalent bonds in the carbon fiber and enhance the amorphization of the fiber surface.

## 1. Introduction

Carbon fiber (CF), due to its high specific strength and modulus, as well as the excellent chemical stability, has been widely used in various fields [1,2,3,4,5,6]. To further extend practical applications of CFs, their surface properties have been improved [7,8] through electrochemical oxidation [9,10,11], plasma treatment [12,13], or heat treatment [14].

Currently, the surface morphology, graphite crystal content, and amorphous carbon ratio of CFs have been successfully characterized by Scanning Electron Microscopy (SEM), Atomic Force Microscopy (AFM), Raman Spectroscopy (RS), and X-Ray Diffraction (XRD) [6,10,15,16,17,18,19,20,21,22,23]. The chemical elements and functional groups on the CF surfaces have been further detected by Fourier Transformation Infra-Red (FTIR) and X-Ray photoelectron spectroscopy (XPS) [24,25,26,27,28]. These techniques reveal that the original CF surface is not smooth, the CF may be cracked in some solutions, and some graphite crystal fragments may fall from the CF surface into the solutions under some extreme conditions. To further illustrate the chemical state of surface-treated CFs, some researchers have also quantified the hydrophilicity through measuring the contact angles of CFs [29]. Meanwhile, a single CF pullout test has been conducted to evaluate the mechanic performance of CFs with different surface microstructures [1,30,31]. These investigations have led to many significantly deepened understandings of the CF surface microstructure and behavior. For example, it is now well-known that the electrochemical oxidation in ammonium salt [9,25] and nitric acid [21] can effectively increase the content of functional groups on the surface of a carbon fiber, which can significantly enhance the tensile strength of a carbon fiber reinforced polymer (CFRP) [32,33,34,35], but too strong oxidation may to some degree damage the CF surface, even though more functional groups have been generated [9,10]. It has also been reported that scattered pits may be formed on CFs at an anodic potential, and the CFs immersed in an strong alkaline NaOH solution can turn brown in color [36] due to some graphite fragments falling off from the CFs into the solution [37]. Apart from the damage in alkaline environments, the electrochemical oxidation in nitric acid can lead to some parts peeling off from CFs as well [21]. Recently, Zhang et. al. [38] illustrated that the cylindrical surface and cross-section surface of a CF could be dissolved differently under strong anodic polarization. The study revealed the anisotropic electrochemical behavior of a CF for the first time and opened up some possible new applications for CF.

Although all the above investigations have clearly indicated that strong polarization can alter the surface state of a CF, no effort has been made to look into the detailed electrochemical activity and surface morphology changes in particular, because most of the studies were simply aimed at improving the adhesion of CFs to their matrix materials [21,25,30,39]. However, in practice, CF may be strongly polarized if it is used as an electrochemical sensor or reinforcement for carbon fiber reinforced polymers (CFRPs) in a service environment with stray current densities [5]. It is quite possible in the testing or service condition, there is a high electric field or current density. Therefore, it is of great scientific interest and practical significance to gain an insight into the electrochemical behavior and morphologic deteriorating process under different polarization conditions.

The study presented in this paper was a continuation of the previous investigation into the anisotropic electrochemistry of CF [38]. The microstructure and composition of a single CF under various polarization conditions were further analyzed, and possible mechanisms responsible for the electrochemical behavior and the polarization induced surface degradation were discussed. The aim of the paper is to validate previous conjectures [38], and deepen the understanding of the electrochemical activity of carbon fiber varying with polarization condition, which is of great significance for carbon fiber sensors in various extreme environments. It is also an innovative perspective to look at the degradation of a CF through an investigation into the electrochemical reactions on the CF surface.

## 2. Materials and Experimental

### 2.1. Materials and Solution

The polyacrylonitrile (PAN)-based CF bundles (Panex 35, 50K) used in this research were purchased from Toray Industries, Inc. (Tokyo, Japan) with an average diameter of 7.2 µm for each single fiber. The CF bundles were cut into 10 cm long prices in the lab. Before test, the carbon fibers were sonicated in acetone for 10 min, rinsed with deionized water, and dried in an oven at 110 °C. The test solution was 3.5 wt.% NaCl.

### 2.2. Electrochemical Measurements

**Figure 1:**
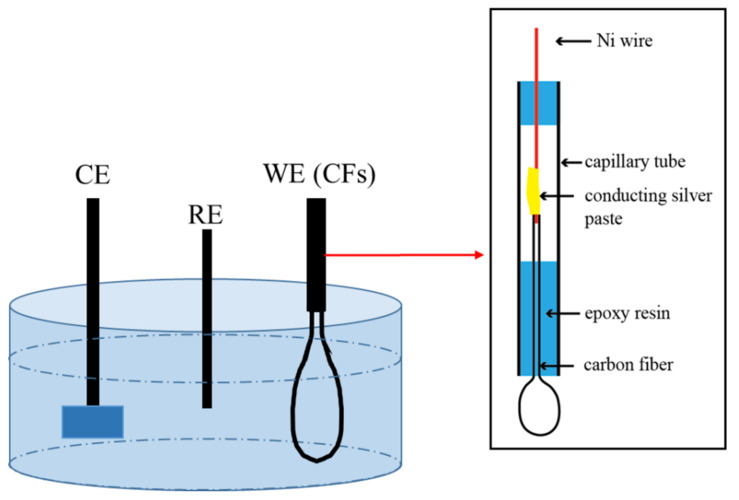
Schematic illustration of the 3-electrode electrolytic cell and the “U”-bent single carbon fiber (CF) electrode [38].

To avoid the exposure of the active end surfaces in solution, a fiber was bent into a shape of letter “U”, and both the fiber ends were sealed in a glass capillary tube, leaving about 4~5 cm of the CF exposed outside the capillary tube as schematically illustrated in Figure 1 [38]. The exposed fiber cylindrical side surface area outside the tube was the working surface of the CF electrode. Electrochemical measurements were conducted using an electrochemical work station (AUTOLAB, PGSTAT302N) in a three-electrode electrolytic cell (see Figure 1), in which the bent single CF was used as a working electrode, a Ag/AgCl electrode filled with saturated KCl as reference electrode, and a 1 cm × 1 cm platinum plate as counter electrode.

The following electrochemical measurements were performed in the 3.5 wt.% NaCl, respectively. The potentiodynamic polarization curve was measured at a scanning rate of 1 mV/s within a potential range from −3.0 V to +3.0 V (vs. OCP) after the single CF was immersed at the OCP for 2 h. The single CF electrode after 2 h immersion in the test solution was also potentiostatically polarized at selected potentials and the potentiostatic current densities were recorded. The electrochemical impedance spectroscopy (EIS) analysis was carried out at the OCP in a frequency range from 10^5^ Hz to 10^−2^ Hz with a perturbation voltage of 10 mV after 2 h immersion, 2 h polarization at a selected potential, and 1 h stabilization at OCP again. The cyclic voltammetry was conducted at a scan rate of 1 mV/s between the OCP and chosen voltages for 3 cycles after the CF was stabilized in the test solution for 2 h. To better reveal the change in surface of the single CF during the cycling experiment and avoid the influences of non-Faradic process and species adsorption/desorption, the scan rate was relatively slow. Most of the electrochemical experiments did not affect the solution in this study. For example, at 3V in the first 0.5 hour, only some bubbles were observed on the Pt surface. Twenty-four hours later, apart from the bubbles, the solution stayed transparently unchanged.

All the electrochemical measurements were performed at room temperature (25 °C) in an air-conditioned lab, and each test was repeated in parallel at least 3 times.

### 2.3. Morphologic Observations

A CF bundle, instead of a single CF, was also potentiostatically polarized at different potentials for 2 h, and the corresponding current densities were recorded. They were cleaned with deionized water and then dried in an oven at 110 °C for morphology observation. The micro morphologies of the CF surfaces within a chosen area of 3 μm × 3 μm before and after polarization were observed under a field emission scanning electron microscope (SEM, SU-70 Hitachi, Tokyo, Japan) at an accelerating voltage of 5 kV and an atomic force microscope (AFM, Dimension Icon Bruker, Billerica, MA, USA) before and after the electrochemical measurements. The changes in CF surface macro morphology and test solution color during the 2 h potentiostatic polarization at +3.0 V vs. OCP were recorded using an optical microscope (Leica DVM6, Wetzlar, Germany).

### 2.4. Microstructure and Composition Characterizations

The CF surface microstructure and composition before and after immersion were also examined by using a Raman spectrometer (Raman, LabRAM HR Evolution Horiba, Montpellier, France) with 532 nm wavelength laser excitation and an X-ray diffractometer (XRD, D8-A25 Bruker, Billerica, MA, USA) with Cu Kα radiation. FTIR spectrometer (FTIR, Nicolet iS50 Thermo Fisher, Waltham, MA, USA) with 4 cm^−1^ resolution was also employed to characterize the surface function groups. These characterizations were carried out on a bundle of the CFs which were cleanly rinsed with deionized water and dried in an oven at 110 °C beforehand.

## 3. Results and Discussion

### 3.1. Electrochemical Behavior and Damage

**Figure 2:**
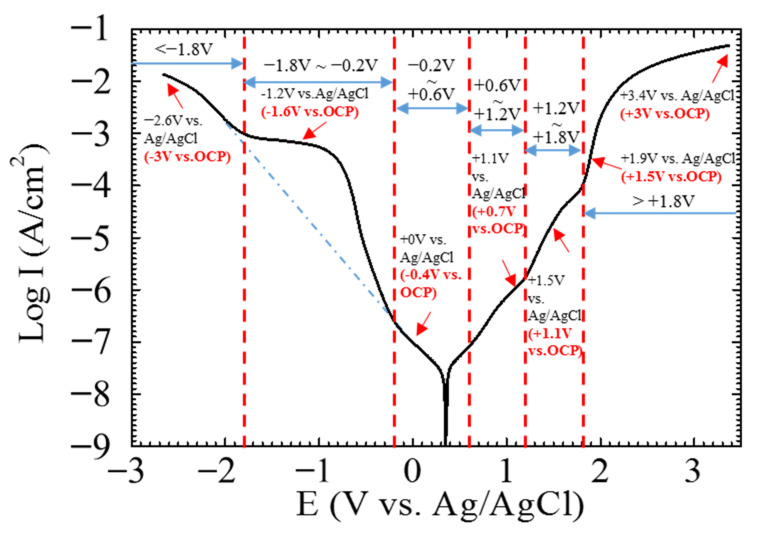
Typical potentiodynamic polarization curve of a CF in 3.5 wt.% NaCl.

The repeated polarization curves of the single CF 3.5 wt.% NaCl solution were not exactly overlapped. A typical polarization curve of the single CF is shown in Figure 2, which shows that the open circuit potential (OCP) of the CF in the 3.5 wt.% NaCl solution was around +0.4 V relative to the Ag/AgCl reference electrode. For simplicity, +0.4 V vs. Ag/AgCl is used as the OCP of the CF in this paper. There are several distinct voltage ranges with a sudden current change on the curve. For example, around the OCP:

−0.2 V~+0.6 V vs. Ag/AgCl or −0.6 V~+0.2V vs. OCP;(1)

in the anodic region:

+0.6 V~+1.2 V vs. Ag/AgCl or +0.2V~+0.8 V vs. OCP,(2)

+1.2 V~+1.8 V vs. Ag/AgCl or +0.8 V~+1.4V vs. OCP,(3)

> +1.8 V vs. Ag/AgCl or > +1.4 V vs. OCP;(4)

and in the cathodic region:

−0.2 V~−1.8 V vs. Ag/AgCl) or −0.6 V~−2.2V vs. OCP, and(5)

−1.8 V vs. Ag/AgCl or < −2.2 V vs. OCP.(6)

The sudden changes in current density in these ranges symbolized different surface states of the CF. To further reveal the electrochemical behavior in these ranges, the CF was polarized at +0.7, +1.1, +1.5, +3.0, −0.4, −1.6, and −3.0 V vs. OCP, which were chosen from each the range, respectively, and the CF surfaces after polarization at the potentials were analyzed. As the electrochemical behavior at the OCP had been analyzed previously [38], it was not measured in this paper again.

**Figure 3:**
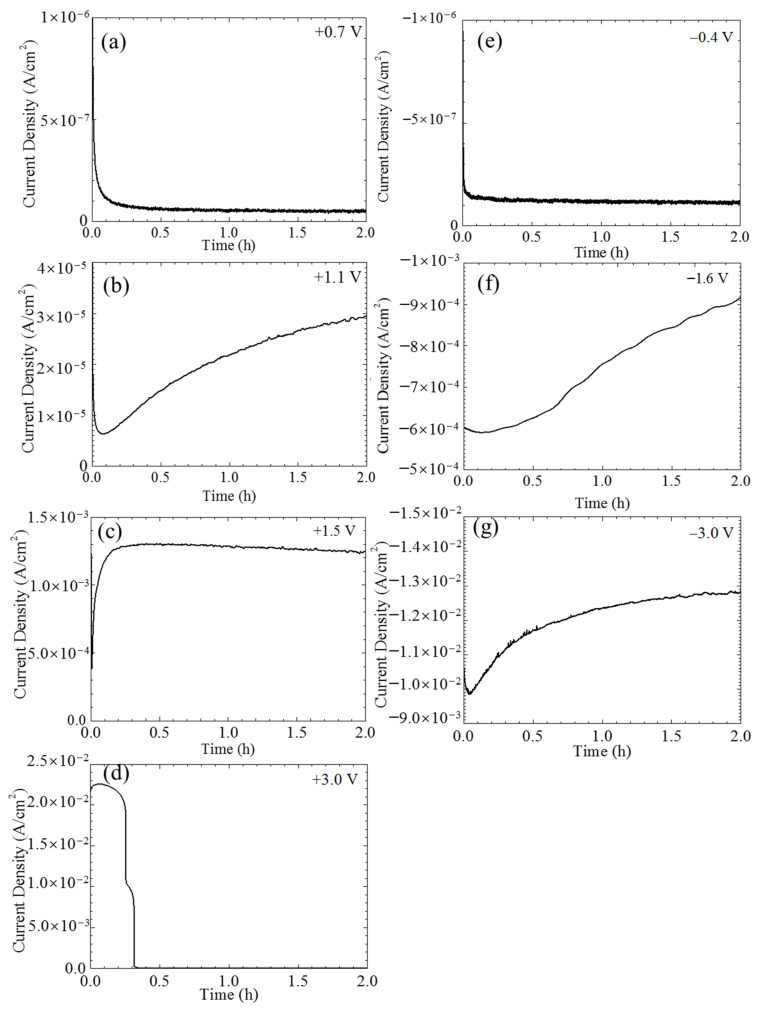
Current densities of a CF varying with time under different potentiostatic potentials: (**a**) +0.7 V vs. OCP, (**b**) +1.1 V vs. OCP, (**c**) +1.5 V vs. OCP, (**d**) +3.0 V vs. OCP, (**e**) −0.4 V vs. OCP, (**f**) −1.6 V vs. OCP, and (**g**) −3.0 V vs. OCP.

The current densities of the single CF electrode varying with time under potentiostatic polarization at different potentials were shown in Figure 3. Certainly, a non-Faradic process could be involved in the recorded current densities in the initial stage. However, such a transient current density usually decays rapidly within seconds. It is not considered in this paper. When the polarization potentials were not too far away the OCP, e.g., at +0.7 and −0.4 V vs. OCP, the current densities initially decreased with time and then gradually reached a stable value around 1 × 10^−7^ A/cm^2^ (see Figure 3a,e). Stronger anodic and cathodic polarization, e.g., at +1.1 V vs. OCP (see Figure 3b) and −1.6 V vs. OCP (see Figure 3f) further sped up the decreasing of the initial current densities, which in the later stage did not reach a stable level, but increased with time instead. The anodic current density in response to an even more positive potential, e.g., +1.5 V vs. OCP, immediately jumped up to a maximum value around 1.3 × 10^−3^ A/cm^2^ and then very slowly decreased with time (see Figure 3c). The variations of the current densities under the extreme potentiostatic conditions, e.g., at potentials ±3.0 V vs. OCP, are shown in Figure 3d,g. Similar to the current density at the cathodic potential −1.6 V vs. OCP, the current density of the CF under −3.0 V vs. OCP polarization also had an initial decrease and then continuously increased with time, but both the decrease and increase were much more rapid, and the increasing rate in the later stage gradually decreased with time; eventually, the absolute current density level was over one order of magnitude larger than that at −1.6 V vs. OCP (see Figure 3d). Interestingly, the current density of the CF at +3.0 V vs. OCP had two sharp drops down from 10^−2^ to 10^−6^ A/cm^2^ (see Figure 3g). This strange behavior was caused by the damage of the single CF electrode, which will be illustrated later. Due to the instability of the electrode, the following electrochemical measurements were not conducted under this polarization condition.

**Figure 4:**
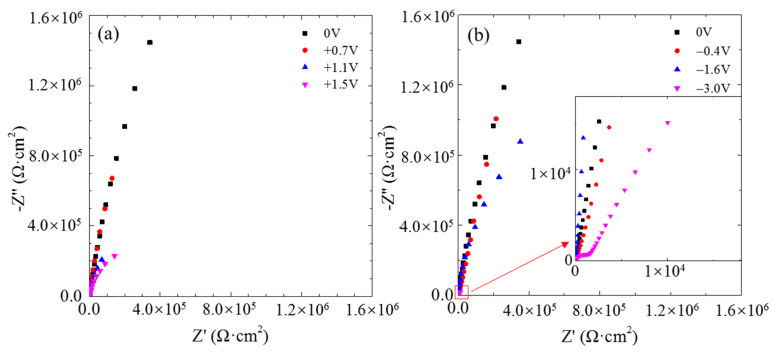
Electrochemical impedance spectra of a CF after 2 h of potentiostatic polarization at different potentials: (**a**) +0.7, +1.1 and +1.5 V vs. OCP, and (**b**) −0.4, −1.6, and −3.0 V vs. OCP.

The typical electrochemical impedance spectra of the single CF electrode at the OCP after 2 h of potentiostatic polarization at different potentials are displayed in Figure 4. Only one incomplete loop appeared in the Nyquist plot at each the anodic polarization potential, indicating a simple electrochemical reaction occurring at the carbon/solution interface. According to the radius of the incomplete loops, the impedance of the CF decreased with increasing anodic potential. After 2 h of cathodic polarization, the impedance also decreased as the cathodic polarization potential became more negative. After the CF was polarized at −3.0 V, it displayed a second capacitive loop in the high frequency range, suggesting that another electrochemical reaction is occurring as well at such a negative potential.

**Figure 5:**
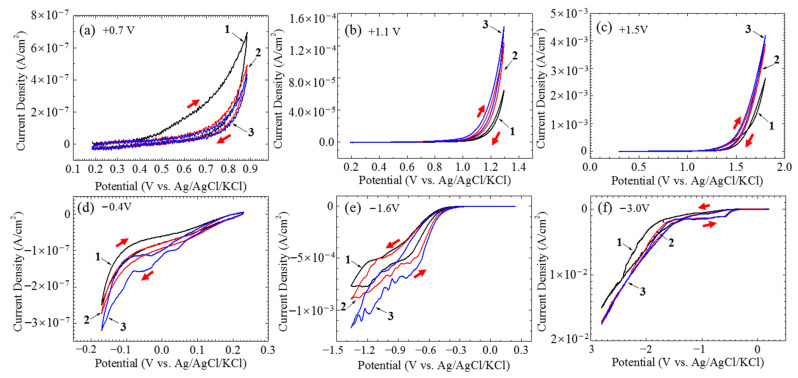
Cyclic voltammograms of a CF from its OCP to different potentials: (**a**) +0.7 V vs. OCP, (**b**) +1.1 V vs. OCP, (**c**) +1.5 V vs. OCP, (**d**) −0.4 V vs. OCP, (**e**) −1.6 V vs. OCP, and (**f**) −3 V vs. OCP.

The cyclic voltammograms of the single CF electrode are shown in Figure 5. The single CF between the OCP and −0.4V vs. OCP had voltammetry current densities decreasing cycle by cycle (see Figure 5a), while from the OCP to the other potentials the voltammetry current densities increased with cycle (Figure 5b–f). The anodic forward scanning current densities were higher than the backward scanning ones. If only scanning between 0 and +0.7V vs. OCP, the differences between the forward and backward scanning current densities were significant (see Figure 5a), while for those more strongly polarized samples, the current density hysteresis loops were much less significant (Figure 5b,c). Similarly, weak cathodic scanning resulted in forward current densities higher than the backward ones (see Figure 5d). However, higher backward current densities were obtained for those samples being strongly cathodic polarized (Figure 5e,f).

**Figure 6:**
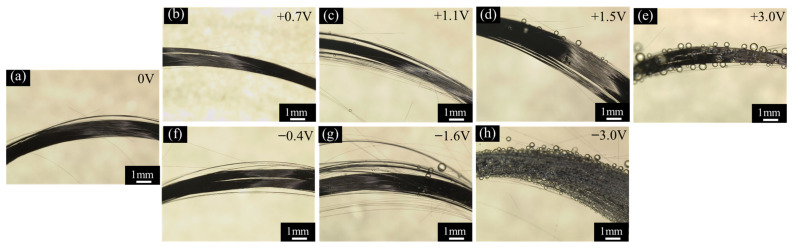
Optical images of CF bundles under polarization at different potentials: (**a**) OCP, (**b**) +0.7 V vs. OCP, (**c**) +1.1 V vs. OCP, (**d**) +1.5 V vs. OCP, (**e**) +3.0 V vs. OCP, (**f**) −0.4 V vs. OCP, (**g**) −1.6 V vs. OCP, and (**h**) −3.0 V vs. OCP.

The optical images of CF bundles under polarization at different potentials are shown in Figure 6. No gas bubble could be detected when the CFs were simple immersed in the test solution (see Figure 6a), polarized at +0.7 V vs. OCP or −0.4 V vs. OCP (see Figure 6b,f). A few small gas bubbles appeared on the surfaces of the CFs when polarized at +1.1 V vs. OCP (see Figure 6c). More and larger gas bubbles were produced on the surfaces when the polarization potentials increased anodically to +1.5 V or cathodically to −1.6 V vs. OCP (see Figure 6d,g). There were a large number of gas bubbles of different sizes formed on the surfaces when the CFs were polarized at +3.0 V or −3.0 V vs. OCP (see Figure 6e,h) in the test solution.

**Figure 7:**
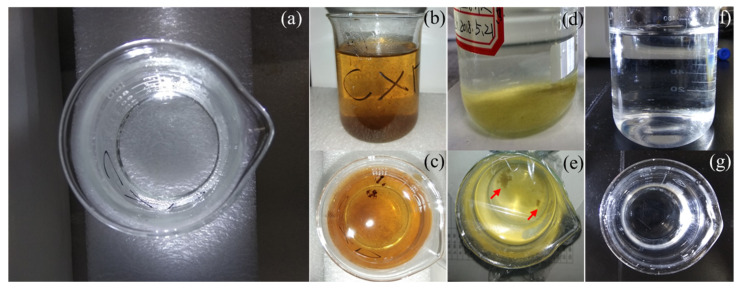
The solution colors (**a**) before and (**b**,**c**) immediately after the CFs being polarized at +3.0 V vs. OCP for 2 h and (**d**,**e**) 24 h later after the polarization; (**f**,**g**) CFs being polarized at −3.0 V vs. OCP for 2 h.

The solution colors before and after 2 h of potentiostatic polarization are shown in Figure 7. The solution was originally transparently clear and colorless before the polarization (Figure 7). However, it turned brown after the polarization at +3.0 V vs. OCP for 2 h (Figure 7b,c). Twenty-four hours later after the polarization, the upper layer of the solution became clear and colorless again while some yellow sediment (indicated by the red arrows in the graph) could be seen at the bottom (Figure 7d,e). However, after the CFs being polarized at −3.0 V for 2 h, the solution color remained clear and unchanged (Figure 7f,g).

### 3.2. Micro Morphology Changes

**Figure 8:**
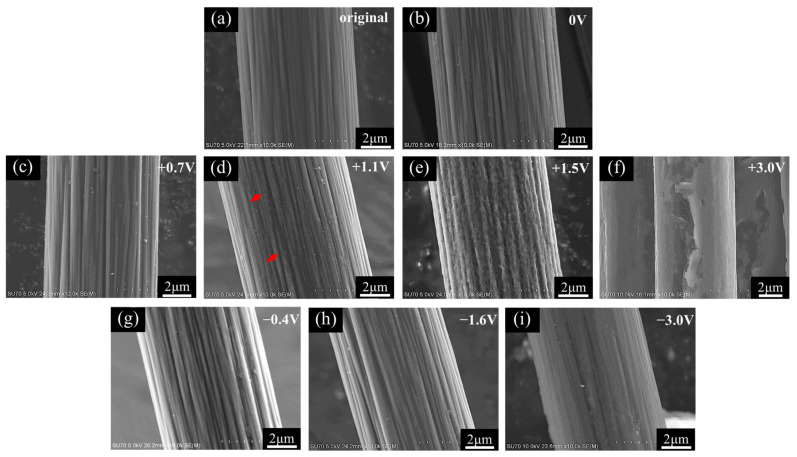
SEM images of the CF surfaces: (**a**) original; (**b**) after 2 h immersion at OCP; and after 2 h polarization at (**c**) +0.7 V vs. OCP, (**d**) +1.1 V vs. OCP, (**e**) +1.5 V vs. OCP, (**f**) +3.0 V vs. OCP, (**g**) −0.4 V vs. OCP, (**h**) −1.6 V vs. OCP, and (**i**) −3.0 V vs. OCP in 3.5 wt.% NaCl.

The SEM images of the CFs before and after 2 h anodic and cathodic potentiostatic polarization are presented in Figure 8. The original surface consisted of numerous distinct longitudinal striations (see Figure 8a). The clear longitudinal striation texture remained unchanged after 2 h immersion in the 3.5 wt.% NaCl (see Figure 8b). Similarly, textured surface was not significantly changed after the CF was polarized at +0.7 V vs. OCP for 2 h (see Figure 8c). When the polarization potential increased to +1.1 V vs. OCP, almost invisible damage occurred randomly in very limited areas of the CF surface as indicated by arrows in Figure 8d. As the potential increased to +1.5 V vs. OCP, the whole surface became rougher but still with a distinguishable striation texture (see Figure 8e). Serious damage occurred on the CF under the extreme anodic polarization at +3.0 V vs. OCP. It appeared that a thick layer of crust was peeled off from the CF in a large area, the diameter of the CF reduced dramatically by half, and the original striation texture was completely wiped out after the 2 h strong anodic polarization (see Figure 8f). Cathodic polarization did not damage the CF surface so badly. There was no obvious change in morphology on the CF surface at −0.4 and −1.6 V vs. OCP (see Figure 8g,h). Even under the extreme cathodic polarization condition at −3.0 V vs. OCP, no peeling off or diameter reduction occurred, but the original striation texture became fuzzy, as if the surface was covered by an opaque film (see Figure 8i).

**Figure 9:**
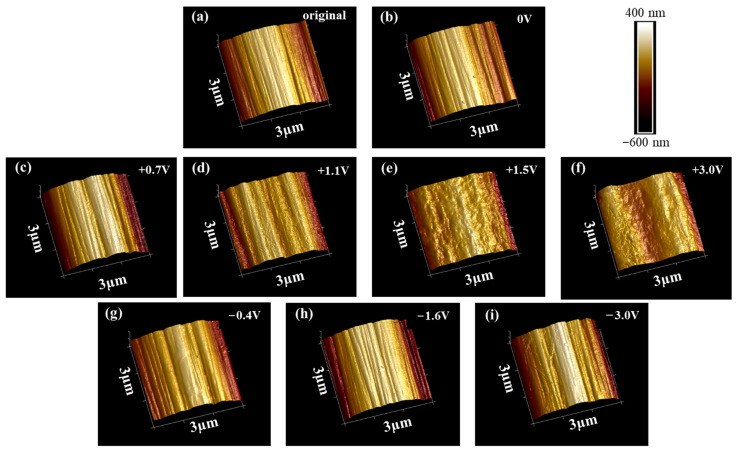
AFM images of the CF surfaces: (**a**) original; (**b**) after 2 h immersion at OCP; and after 2 h polarization at (**c**) +0.7 V vs. OCP, (**d**) +1.1V vs. OCP, (**e**) +1.5 V vs. OCP, (**f**) +3.0 V vs. OCP, (**g**) −0.4 V vs. OCP, (**h**) −1.6 V vs. OCP, and (**i**) −3.0 V vs. OCP in 3.5 wt.% NaCl.

To avoid possible dehydration of some surface compounds and interaction of electron beam with the surface products on the CF in high vacuum during SEM examination, both of which might significantly alter the surface morphology of the immersed and polarized CF, AFM was employed to characterize the surface micro morphologies of the CF. The results from the selected 3 × 3 μm area (see Figure 9) show many surface features similar to those revealed by SEM. The distinct striations running longitudinally along the CF were clearly visualized on the original surface (see Figure 9a). Such morphology did not change at all after 2 h immersion at OCP (see Figure 9b) and +0.7 V vs. OCP (see Figure 9c). The distinguishable striation text on the CF surface after 2 h polarization at +1.1 V vs. OCP became slightly rougher (see Figure 9d). At +1.5 V vs. OCP, the surface was much rougher, full of bumps, and the striation texture almost disappeared (see Figure 9e). At the extreme anodic potential +3.0 V vs. OCP, no striation could be seen on the surface any more, but a large groove along the CF body (see Figure 9f). If the CF was cathodically polarized at −0.4 and −1.6 V vs. OCP for 2 h, the original surface morphology kept unchanged (see Figure 9g,h). After 2 h of the extreme cathodic polarization at −3.0 V vs. OCP, a slightly blurred striation texture could still be seen on the CF surface (see Figure 9i).

### 3.3. Surface Physical Chemical States

**Figure 10:**
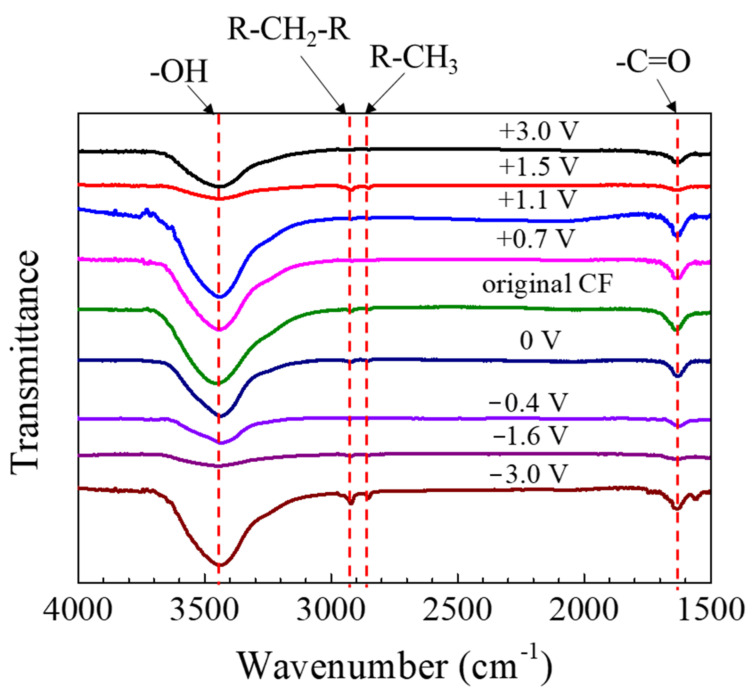
The FTIR spectra of the CFs before and after 2 h of polarization at different potentials vs. OCP.

The FTIR spectra of the CF after 2 h of potentiostatic polarization are shown in Figure 10. The peak around 3500 cm^−1^ could be assigned to -OH; the one at 1635 cm^−1^ should correspond to -C=O, and those at 2922 cm^−1^ and 2850 cm^−1^ could be attributed to the stretching vibration of -CH_2_- and -CH_3_ groups, respectively [20,25,40]. The peaks did not shift when the polarization potential changed, although the peak height varied.

**Figure 11:**
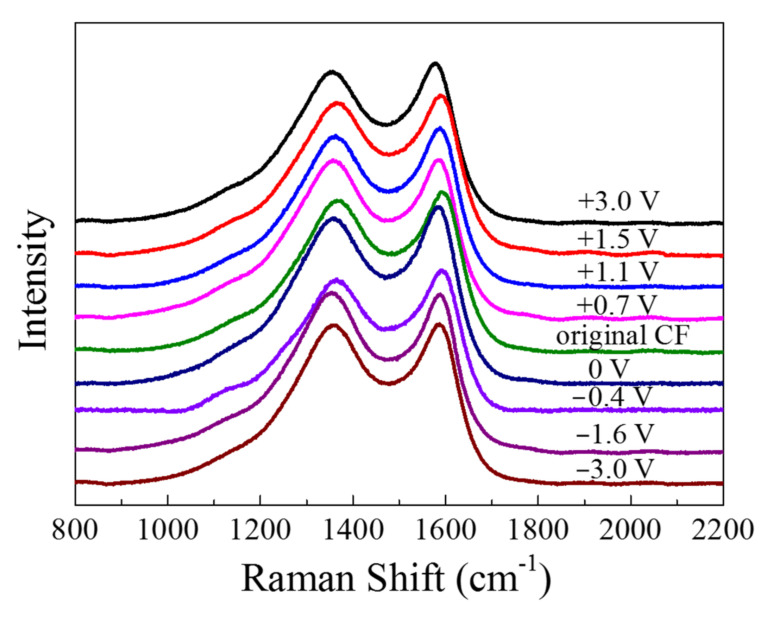
Typical Raman spectra of the CFs before and after 2 h of polarization at different potentials vs. OCP.

The Raman spectra are displayed in Figure 11. There were two major peaks in the spectra: The peak at 1360 cm^−1^, which is known as “D band”, was associated with the disordered carbonaceous structure on the CF surface, while the “G band” at 1580 cm^−1^ represented the ordered graphitic structure [20]. The “D band” and “G band” of the CF became sharper and shifted to left after anodic and cathodic polarization. By fitting the spectroscopy of fiber with Gauss–Lorentz function, the “R” value (=I_D_/I_G_) representing disorder degree could be obtained (see Table 1). It can be seen that all the polarized CF samples had a *R* value larger than the original CF before and the CF immersed in the solution for 2 h, indicating that both the anodic and the cathodic polarization could lower the content of graphite phase or enhance the disordered carbonaceous structure on the surface. As the anodic polarization potential became more positive from +0.7 to +3 V vs. OCP, the *R* increased from 2.069 to 2.507. Vice versa, as the polarization potential changed from −0.4 V to more negative −3 V vs. OCP, the *R* value also became larger from 2.057 to 2.420.

**Figure 12:**
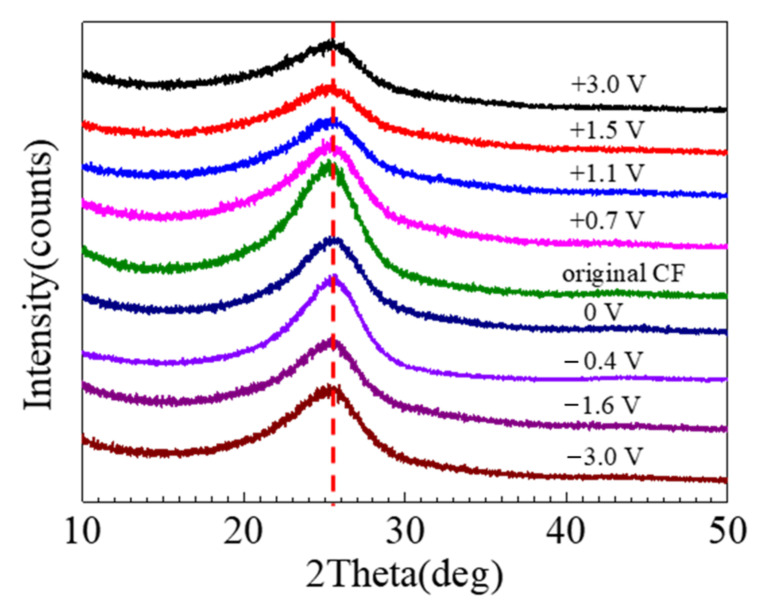
XRD spectra of the CF before and after 2 h polarization at different potentials.

The XRD spectra of the CFs are shown in Figure 12, which has a bump centered at 25.5°, corresponding to the graphitic crystallographic plane (002) [18]. In the XRD results, the differences caused by the 2 h polarization were insignificant. The crystallographic plane spacing d_(002)_ could be estimated according to Bragg and Scherer equation, which in comparison with the theoretical value can be employed to indicate the degree of graphitization [16]. It appeared that the XRD peak decreased after immersion, and the decrease was more significant after strong anodic polarization, while the influence of cathodic polarization was insignificant.

### 3.4. Electrochemical Activity

Carbon fiber contains over 90% carbon atoms in a crystalline structure similar to graphite, in which the carbon atoms or carbon atom groups along the fiber in the longitudinal direction are connected together by covalent bonds in chains, and these carbon chains are held together through Van der Waals force in the radial direction [41]. The damage to a CF caused by polarization should result from breakdown of the chemical and physical bonds between and in the chains [22], which could be conjectured from electrochemical measurements.

The three voltage ranges in the anodic and also in the cathodic regions respectively on the polarization curve of the single CF (see Figure 2) represent different electrochemical processes and surface activities. 

In the relatively low potential range −0.2 V~+0.6 V vs. Ag/AgCl or −0.6 V~+0.2 V vs. OCP, e.g., at 0 V vs. Ag/AgCl or −0.4 V vs. OCP, the current densities were lower than 1 × 10^−7^ A/cm^2^, and the impedance was very high (see Figure 2, Figure 3e and Figure 4), which meant that the CF just performed like an inert resistance. It simply offered a surface area for oxygen production (4OH^−^ → O_2_ + 2H_2_O + 4e^−^) and reduction (O_2_ + 2H_2_O + 4e^−^ → 4OH^−^). These anodic and cathodic reactions were very weak, and thus, no gas bubble was observed on the CF surface (see Figure 6a,f). The other reactions, including the anodic and cathodic hydrogen reactions (H_2_ → + 2H^+^ + 2e^−^ and 2H^+^ + 2e^−^ → H_2_), were undetectable either due to the high over-potentials. The weak anodic or cathodic polarization could not change the surface morphology of the CF (see Figure 8b,g and Figure 9b,g). It should be noted that in this potential range, the oxygen reduction was very slow and was not be limited by the oxygen diffusion step in the solution. Hence, no Warburg characteristic was detected in the EIS (see Figure 4). The only abnormal result was the voltammetry current densities increasing cycle by cycle (see Figure 5d), which contradicted the potentiostatic current density quickly decreasing with time and reaching a stable level (see Figure 3d). This is probably because some of the oxygen at the CF/solution interface was consumed and the oxygen reduction slowed down reaching a new steady state rapidly (see Figure 3d). The increasing voltammetry current densities with cycling (see Figure 5d) cannot be easily interpreted. There is no certainty if more electrochemically active sites could be generated by cycling potentials than a constant potential. The backward scanning current densities relatively lower than the forward scanning ones might result from the back charged non-Faradic current density.

Beyond this potential range, the anodic and cathodic transitions occurred on the polarization curve. Anodically, the potential range from +0.6 to +1.2 V vs. Ag/AgCl or from +0.2 to +0.8 V vs. OCP (see Figure 2) might result from oxidation of some impurities on the CF surface. The impedance in this potential range, e.g., at +1.1 V vs. Ag/AgCl or +0.7 V vs. OCP, decreased (see Figure 4), which was consistent with the increased current densities compared with those in the potential range around the OCP (see Figure 2). However, oxygen evolution was still relatively insignificant, and no visible bubble formed on the CF surface (see Figure 6b). The consumption of the impurities could result in a decrease in potentiostatic current density, e.g. at +0.7 V vs. OCP, with time (see Figure 3a) and in voltammetry current densities cycle by cycle (see Figure 5a). This conjecture could be supported by the decreased backward scanning current densities compared with the forward current densities in this potential range. Obviously, the dissolution of the trace amounts of impurities could not cause an obvious morphologic change on the CF surface (Figure 8c and Figure 9c).

Further increasing the polarization potential led to another sudden change in current density in the range of +1.2~+1.8 V Ag/AgCl or +0.8 to +1.4 V vs. OCP (see Figure 2). The potentiostatic current density in this potential range, e.g., at +1.5 V vs. Ag/AgCl or +1.1 V vs. OCP, kept increasing with time in the later stage (except the initial decreasing non-Faradic process) (see Figure 3b), which could be associated with oxygen evolution [38], as a few gas bubbles were formed on the CF surfaces (see Figure 6c), i.e., the oxygen evolution started to dominate the anodic process on the CF surfaces. The EIS result (Figure 4) showed that the impedance in this potential range further decreased, which also indicated the single CF surface damaged. After 2 h of potentiostatic polarization at +1.5 V (Ag/AgCl), some deeper grooves were formed on the CF surface and the surface became rougher (Figure 8d and Figure 9d), providing a larger surface area for the oxygen evolution. The generated bubbles covered some area of the CF surface and thus to some extent retarded the increasing trend of the current density (see Figure 3b). The CV result (Figure 5b showed that the reaction on the CF surface became faster and faster and cycle by cycle, in consistence with the increasing potentiostatic current density with time (see Figure 3b). The voltammetric current hysteresis loop in this potential range became less significant (see Figure 5b), probably due to the non-Faradic current density relatively negligible compared with the Faradic current density in this case.

The last anodic current transition started from +1.8 V vs. Ag/AgCl or +1.4 V vs. OCP (see Figure 2). When the polarization potential was more positive than the transition point, the current density continuously increased with potential. The increased polarization current density was in coincidence with the further decreased impedance at +1.9 V vs. Ag/AgCl or +1.5 V vs. OCP (see Figure 4). Correspondingly, the potentiostatic current density rapidly increased with time to a very high level (see Figure 3c). These increasing current density, as well as the increased polarization current density and decreased impedance, could be attributed to the CF surface roughening (see Figure 8e and Figure 9e), which was also responsible for the current densities increasing with cycling (see Figure 5c). Due to the relatively insignificant non-Faradic process, the CV hysteresis behavior was insignificant, too. Interestingly, at this potentiostatic potential, the current density after reaching the maximum slowly decreased with time (see Figure 3c). The slow decreasing current density could not be interpreted by the gradually or slightly roughened CF surface (see Figure 8e and Figure 9e). It was noticed that more and larger gas bubbles were generated on the CF surface at this potential (see Figure 6d). Since the solution contained chlorides, under such a strong anodic polarization, chlorine might also be produced (2Cl^−^ → Cl_2_ + 2e) in addition to the vigorous evolution of oxygen. The newly generated oxygen atoms and chlorine might interact with C atoms and break the C-C bonds in the CF, resulting in cracks in the CF, and eventually even some fragments peeling off from the CF. The cracks or the fragments before peeling off may to some degree separate the CF surface from the solution and slightly reduce the current density in the later stage (see Figure 3c). 

**Figure 13:**
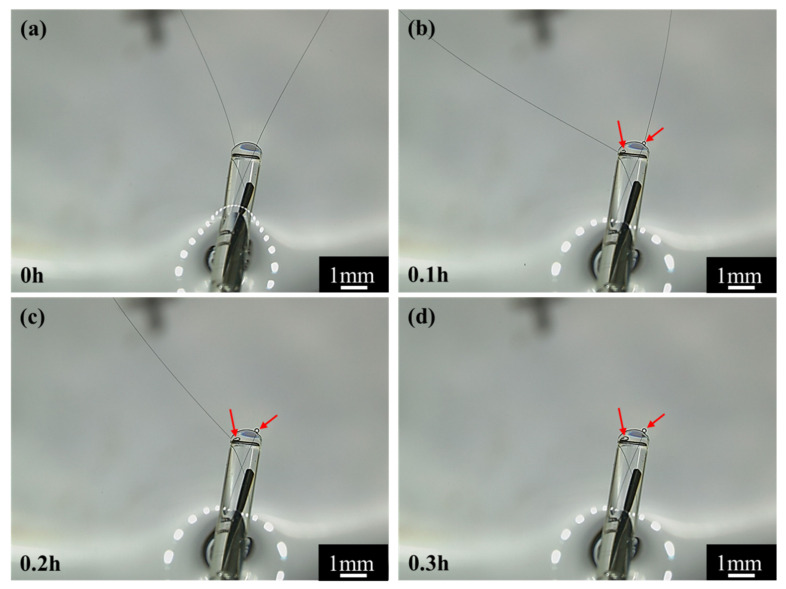
The optical images of CFs under +3.0 V (vs. OCP) polarization at different times. (**a**) 0 h, (**b**) 0.1 h, (**c**) 0.2 h and (**d**) 0.3 h.

To validate the conjecture regarding the cracking and peeling of CF, the anodic polarization potential was further increased to +3.4 V vs. Ag/AgCl or +3.0 V vs. OCP. Under such an extreme anodic condition, the cracking and peeling effect would be strong enough to produce some visible changes. The most astonishing observation was the current density suddenly dropped to 0 in 0.25 h (see Figure 3d). Figure 13 shows that the changes of the single CF during the potentiostatic polarization at this high potential. In the first 0.1 h when the current density was around 0.022 A/cm^2^ (see Figure 3d), many gas bubbles were generated on the CF surface (Figure 13b). The current density dropped to 0.01 A/cm^2^ in about 0.2 h (see Figure 3d) when the CF was broken at one end (Figure 13c). The whole exposed CF surface was still conductive, as it was still electronically connected through the other end. The drop of current density down to 0 in 0.2~0.3 h corresponded to the CF broken at the other end, too (Figure 13d). In this case, the originally exposed part of the single CF was completely disconnected. It can be expected that if the breakdown took place on many CF filaments in the same time, then a large number of CF fragments would be formed, which could significantly influence the transparency and color of the solution. This was supported by the CF bundle polarized at +3.4 V vs. Ag/AgCl or +3.0 V vs. OCP. At this extreme anodic potential, lots of oxygen (including chlorine) bubbles appeared on the CFs (see Figure 6e). After 2 h, the test solution turned brown (see Figure 7b,c), and yellow sediment could be obtained at the bottom of the solution after 24 h (see Figure 7e,f), indicating a large amount of CF fragment deposition had been accumulated in the baker. Since similar fragments peeling off from CFs have been reported during the electrochemical oxidation in nitric acid [21], it is not surprising that such damage could also occur in the NaCl solution under the strong anodic polarization. The SEM and AFM images under the extreme anodic polarization condition provided additional evidence for the damage (Figure 8f and Figure 9f).

On the cathodic branch of the polarization curve, the first transition started at −0.2 V vs. Ag/AgCl or −0.6 V vs. OCP. In the range from −0.2 V vs. Ag/AgCl or −0.6 V vs. OCP to −1.8 V vs. Ag/AgCl or −2.2 V vs. OCP, the current density first increased rapidly and then slowly (see Figure 2). At −1.2 V vs. Ag/AgCl or −1.6 V vs. OCP in this potential range, the potentiostatic current density was increasing most of the time (Figure 3f), and the impedance was much smaller than that near the OCP (see Figure 4). Both the current density and impedance behaviors were consistent with the increasing voltammetry current densities cycle by cycle (see Figure 5e). They could result from the occurrence of more active cathodic hydrogen evolution in addition to the relatively weak oxygen reduction, which dominated the cathodic process under this cathodic polarization condition, as several gas bubbles could be seen on the single CF (see Figure 6e). The cathodic hydrogen evolution reaction was facilitated dramatically when the cathodic polarization was more negative than its over-potential (perhaps around the transition point). Because of the hydrogen evolution, the solution was stirred, which sped up the oxygen reduction. Hence, the backward scanning current densities were higher than the forward scanning current densities (see Figure 5e). At more cathodic potentials in the range, too many hydrogen bubbles stuck on the CF surface would retard the increasing trend of current density (see Figure 2). The hydrogen evolution reaction could not dissolve or damage the CF as effective as the oxygen reduction. Hence, the CF surface morphology did not change evidently (see Figure 8h and Figure 9h).

When the cathodic polarization was more negative than −1.8 V vs. Ag/AgCl or −1.4 V vs. OCP, the current density jumped up again (see Figure 2). The potentiostatic current density at −2.6 V vs. Ag/AgCl or −3.0 V vs. OCP in this extreme cathodic condition continuously increased with time (see Figure 3g), which was consistent with the increasing voltammetry current densities with cycling (see Figure 5f). The remarkable increase in cathodic current density over the transition point could not be attributed to suddenly accelerated cathodic hydrogen evolution, as it is normal that more hydrogen gas bubbles was generated on the CF surface (Figure 6h) at a more negative potential. Therefore, it is postulated that the oxygen reduction became more intensive than the hydrogen reaction. In fact, hydrogen evolution could produce a large number of bubbles stuck on the CF surface, which could to some degree retard its further enhancement, while the significantly increased local pH value of the solution adjacent to the CF surface could evidently accelerate the oxygen reduction. The stronger oxygen reduction and hydrogen evolution in this potential range would yield deceased impedance (see Figure 4). Since the suddenly increased cathodic current density was caused by significantly accelerated oxygen reduction, the solution stirring adjacent to the CF surface was not dramatically accelerated. Hence, the CV hysteresis become insignificant again (see Figure 5f). However, the appearance of a high frequency capacitive loop in this condition was unexpected during the EIS measurement. The change in the CF surface morphology revealed by SEM and AFM (see Figure 8i and Figure 9i) might offer a clue to the interpretation of the additional capacitive EIS behavior. The SEM and AFM images indicate that a thin and loose film might have been formed on the CF, which blurred the original surface texture feature. This film could be a non-protective layer deposited at high alkalinity, perhaps consisting of some impurities originally dissolved from the CF in the potential range near the OCP. It could exhibit a small capacitive loop in addition to the large capacitive loop associated with the electrochemical activity of the CF. 

**Figure 14:**
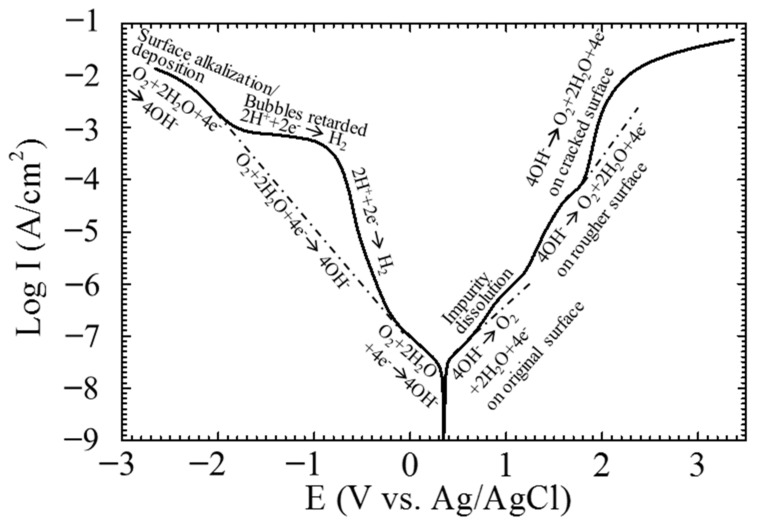
Summary of the electrochemical reactions on the CF surface.

The above analyses regarding the electrochemical behavior of the single CF are summarized in Figure 14.

### 3.5. Electrochemical Damage

The above results indicate that electrochemical polarization could significantly change the surface micro morphology of a CF, which confirmed the previous experimental observations [38]. Obviously, the surface morphological changes can physically lead to different electrochemical activities or behaviors of the CF. It is more important to further understand the cause of the electrochemical changes in molecular level. 

According to the FTIR (see Figure 10), the surface of the CF bundle should contain functional groups -OH, -CH_2_-, -CH_3_ and -C=O [20]. Both the anodic and cathodic polarization at different potentials did not change these surface chemical groups. Even under the extreme polarization conditions at ±3 V vs. OCP when the CF surface micro morphology had been noticeably changed, similar functional groups still remained on the CF surface. In this case, the weak -CH_2_- and -CH_3_ peaks only varied slightly, but the variation was not significant enough to be meaningful. The result suggested that the functional groups on the CF surfaces might be formed immediately again in the newly exposed surface areas of the CF bundle after cracking or peeling-off.

In the Raman spectra (see Figure 11), the G band of graphite around 1580 cm^−1^ and the D band around 1360 cm^−1^ for disordered finite-sized microcrystalline carbons [42,43,44] change with the polarization potential. Both the “G” and the “D” bands can generally be attributed to the graphitic sp^2^ bonded carbons. The *R* ratios of I_D_/I_G_ for all the samples, obtained by curve fitting according to the Gauss–Lorentz function, are shown in Table 1. An increased I_D_/I_G_ ratio means that the percentage of graphite microcrystalline content on the surface decreases and the amount of disordered carbons increases. Hence, the results listed in Table 1 indicate that the disorder degree of the CF bundle increased significantly when the CF bundle was anodically or cathodically polarized, suggesting that the polarized CF bundle became more disordered, containing more amorphous carbonaceous substances [17]. This was probably due to the original covalent bonds in the graphite structure being broken or at least seriously distorted by the polarization [22] and some new covalent bonds being formed among the disordered carbon atoms in each the graphite layers. 

A chemical change is normally accompanied by physical damage [22]. For a CF, the physical damage can be mechanical cracking and peeling. The postulated bond changes by strong polarization may be symbolized by a change in crystalline microstructure of the CF bundle. The XRD is a powerful tool to detect the lattice parameters for a crystal. For graphite, the XRD peak around 25.5° corresponds to its crystal plane (0 0 2), whose nature interlayer spacing is 3.354Å [45,46]. In theory, a variation in graphitization degree will lead to a change in the peak. It is interesting that the peak location did not noticeably shifted, but the peak height decreased after the polarization (see Figure 12), implying that the increased number of the disordered C atoms led to a decreased amount of the crystal graphite in the CF bundle, but the graphite crystal plane distance was not affected by the decreased graphitization degree. This is understandable, as disordered C atoms could be in the sites either nearer or farther than the typical graphitic spacing.

It is quite likely the dissolution, cracking or peeling-off of the CF under strong anodic polarization could be associated with the disordering or amorphization of C atoms because these atoms severely deviated from their crystalline lattice were more active and could easily react with new generated O atoms. Under cathodic polarization, neither the generated hydrogen could further reduce the C atoms, nor the produced hydroxyls were reactive with the C atoms. Therefore, the cathodic polarization was much less detrimental to the CF. The electrochemical activity enhanced by the disordering or amorphization of the C atoms in the CF may also help understand the dramatically accelerated oxygen evolution by strong anodic polarization and the oxygen reduction taking over the hydrogen evolution on the alkalized CF when the CF was strongly cathodically polarized. 

It is not clear how the polarization affected the graphitization degree in this study, which needs further investigations in future.

## 4. Summary

The surface state and electrochemical activity of a single CF can be changed by polarization. When a CF is naturally immersed or weakly polarized around its OCP, oxygen generation and reduction occur. Apart from the oxygen generation under anodic polarization, the CF may be dissolved. Under strong anodic polarization, the CF can even be peeled off and cracked. The roughened surface and new exposed surface in the peeled and cracked areas of the CF can significantly enhance the oxygen generation reaction. The cathodic process is a competition between oxygen reduction and hydrogen evolution. When cathodic polarization exceeds the hydrogen over-potential, hydrogen reduction dominates the electrochemical process. At a more negative potential, the oxygen reduction becomes a dominating reaction again. 

Both the anodic and cathodic polarization can lead to a decrease in graphitization of CF surface. The increased disordering or amorphization of the C atoms in the CF by strong polarization may be responsible for the accelerated dissolution, peeling-off and cracking of the CF under strong anodic polarization.

An important implication from this study is that the application of carbon fiber should better be limited in an environment without interference of strong electric field or high current density. A potential or current sensor can be made of carbon fibers, but its potential or current range should be controlled carefully. On any carbon fiber reinforced polymer or other composites containing carbon fiber bundles, the carbon fiber exposed areas, such as the cut-edge, the bolt hole, or an accidental scratch, should be carefully protected if a stray current in the environment is unavoidable. 

## Figures and Tables

**Table 1:** The R value or ratio of I_D_/I_G_ obtained from CFs after 2 h of polarization at different potentials.

Sample	−3.0 V vs. OCP	−1.6 V vs. OCP	−0.4 V vs. OCP	0 V vs. OCP	Original CF	+0.7 V vs. OCP	+1.1 V vs. OCP	+1.5 V vs. OCP	+3.0 V vs. OCP
I_D_/I_G_	2.420	2.214	2.057	2.042	2.040	2.069	2.152	2.270	2.507

## Data Availability

The data presented in this study are available on request from the corresponding author. The data are not publicly available due to that the data may be further processed for other purposes.
